# Deriving lessons learned from monitoring adaptation activities in projects under the EU mission on adaptation

**DOI:** 10.12688/openreseurope.17372.2

**Published:** 2025-11-12

**Authors:** Stephanie Bilgram, Carla Klusmann, Christian Kind, Elisa Andreoli, Chiara Castellani, Dimitris Kofinas, Jan Cools, Antonio Trabucco, Chrysi Laspidou

**Affiliations:** 1 adelphi research gemeinnutzige GmbH, Berlin, Berlin, 10559, Germany; 2 Thetis S.p.A., Venezia, 30122, Italy; 3 Civil Engineering Department, University of Thessaly, Volos, 38334, Greece; 4 Institute of Environment and Sustainable Development, University of Antwerp, Antwerpen, 2020, Belgium; 5 Department of Engineering, University of Antwerp, Antwerpen, 2020, Belgium; 6 Fondazione Centro Euro-Mediterraneo sui Cambiamenti Climatici, Lecce, 73100, Italy

**Keywords:** Monitoring, climate resilience, climate adaptation, indicators, metrics

## Abstract

Actions to strengthen climate resilience are gaining more traction. In order to ensure effective adaptation, it is important to monitor the outcomes and impacts of these actions. However, there are numerous challenges and a multitude of approaches when it comes to monitoring adaptation to climate change. This paper addresses challenges and lessons learned in setting up mechanisms for monitoring climate resilience and adaptation projects. Drawing from three EU Horizon 2020 projects under the EU Mission on Adaptation to Climate Change, it synthesizes insights to support future initiatives in their monitoring endeavors for other projects to learn from. Findings, acquired through workshops with experts, highlight four key challenges and the projects’ learnings: the challenge of tailoring global frameworks to local needs, data availability and evaluation of data, interdisciplinary collaboration in monitoring, and stakeholder engagement for monitoring endeavors.

## Introduction

Monitoring climate resilience is gaining importance on the global and on the European agenda especially as its significance is highlighted in the EU Strategy on Adaptation to Climate Change. The strategy's objective is to engage more regions in adaptation initiatives and to facilitate the transfer of locally developed solutions to a wider regional and national context ( European Commission, Directorate-General for Climate Action, 2021b). The EU Mission on Adaptation to Climate Change builds upon this strategy and was adopted to support regions across Europe in achieving climate resilience by 2030. Under the umbrella of the mission, there are a number of applied research projects that assist regions in implementing adaptation activities and monitoring progress towards enhanced climate resilience ( European Commission, Directorate-General for Climate Action, 2021a).

Monitoring and subsequent evaluation are the fundamental requirements for understanding which adaptation actions and policies prove successful and for comprehending how climate resilience changes over time. Additionally, sound monitoring can contribute to the effectiveness of adaptation measures, can strengthen accountability and allows tracking progress as well as outcomes and impacts. The value of using common conceptual frameworks for tracking governmental efforts towards global climate change adaptation has been highlighted, also revealing common challenges ( Berrang-Ford *et al*., 2019; Tompkins *et al*., 2018). Furthermore, monitoring and evaluation at the regional level allows drawing lessons learned from implementing transformative actions for risk reduction and resilience enhancement. Given that various sectors, such as transport, water and healthcare, often fall under regional government jurisdiction, the importance of monitoring at the regional level cannot be overstated ( Setzer *et al.*, 2020).

This is why under the Mission on Adaptation, the Regional Adaptation Support Tool (RAST) was developed, which emphasizes that monitoring should gauge progress with respect to: reducing climate impacts, reducing risks and vulnerabilities and increasing adaptive capacity, meeting adaptation priorities and addressing barriers to adaptation. However, designing a monitoring (and evaluation) framework that comprehensively measures development towards climate resilience can be challenging for various reasons and there is not one approach that fits all contexts to monitor and evaluate adaptation actions taken ( ClimateADAPT, 2023; New *et al*., 2022). The differing impacts on various fields, such as infrastructure, economy, communities, institutions, ecosystems etc., outline the different dimensions that a monitoring approach has to cover, which is only one reason why multiple approaches are needed.

Therefore, this document presents a preliminary collection of challenges and lessons learned derived from three individual interviews and three joint workshops with three EU Mission Adaptation projects (ARSINOE, IMPETUS, TransformAr) that have started developing and implementing adaptation measures in vulnerable EU regions. As the projects are still ongoing until 2025, the following are solely initial observations and learnings focusing on the preparatory and early implementation phase of monitoring activities.

The three projects follow very different approaches which make them interesting to consider when looking at monitoring approaches. Despite their differences, which is described in the next chapter, the researchers were able to identify four crosscutting themes that affect all three projects: : *(i) tailoring global frameworks to regional/local needs, (ii) data availability, applicability, and evaluation, (iii) interdisciplinarity in climate adaptation and (iv) stakeholder engagement*. The project REGILIENCE initiated the gathering of lessons learned from the three projects via joint workshops with experts from the projects to support future mission projects in their monitoring endeavors. The document thus functions as an information source for any project and region conducting monitoring (and evaluation) activities. It provides inspiration and preemptive considerations for future Mission projects and is intended to help circumvent previously identified difficulties to avoid common pitfalls. Eventually, this can contribute to a more efficient and streamlined use of project resources.

## Methodology

This study employed a multi-step approach to gather insights and challenges faced by three distinct projects: ARSINOE, TransformAR and IMPETUS. The data collection process began with three workshops involving project partners engaged in the development of monitoring frameworks. The workshops aimed to develop a common understanding of monitoring practices and an exchange on initial approaches and common challenges in the projects. Following the workshops, semi-structured interviews were conducted with representatives from each project. The interviews aimed to assess the current status quo of monitoring activities, identify recent developments and challenges, and extract lessons learned from the development stages of their respective monitoring schemes. This approach allowed for detailed and nuanced information regarding the challenges and learnings experienced by each project in their early stage of developing monitoring approaches.

Participants were selected based on their roles and responsibilities in the projects’ monitoring activities. The information collected from the workshops and interviews were transcribed, and then thematically clustered and analyzed to identify key themes and patterns. This synthesis was then verified with the interviewees to ensure that information was taken up correctly. While the interviews and workshops provided valuable insights, it is important to note limitations such as the small sample size and potential subjectivity in qualitative analysis.

## About the three projects

### ARSINOE project and its approach to monitoring climate resilience

ARSINOE aims to leverage innovative, cross-sectoral climate change adaptation solutions, as well as leveraging regional databases and climate and impact simulations. The project involves collaborations among various stakeholders across the quintuple innovation helix ( Carayannis *et al.*, 2012), including academics, authorities, municipal companies, agriculture, forestry, water and environmental protection groups, technology providers, urban planners, and citizens. Through a living lab approach, the initiative fosters a shared understanding of the impact of climate change. ARSINOE develops data analysis tools and models to facilitate the design of adaptation strategies and measures. Hence, it contributes to resource management, energy, water and food security, and preserving ecosystem functions and services.

***Monitoring framework***

ARSINOE’s monitoring work is based on the Sendai Framework and the Sustainable Development Goals (SDGs) as overarching frameworks. SustainGraph ^1^ is an innovative tool and major outcome of ARSINOE that monitors resilience on a broader level – to the extent where resilience relates to sustainable development – and monitors progress towards achieving SDG-targets. This tool acts as a unified knowledge source, leveraging graph databases and machine learning techniques for data population, knowledge production, and analysis. It maximizes the use of available data, ensuring openness and interoperability with existing databases and Application Programming Interfaces. The SustainGraph facilitates participatory modeling and analysis processes for socio-environmental and socio-ecological systems ( Fotopoulou *et al.*, 2022). It aligns with the principles of a Systems Innovation Approach ^2^ ( Schuurman *et al.*, 2023). Additionally, the project intends to develop a Multi-System Dynamic Modelling Framework for Resilience Assessment, by the end of the project, that will integrate the various modelling subsystems that have been developed in various academic disciplines and use discipline-specific methods, tools and techniques, enabling transdisciplinary modelling of both natural and man-based systems.

***Monitoring at demo-site level***

ARSINOE is also employing a bottom-up approach for demo site-specific monitoring. This is achieved by establishing “living labs”. Stakeholders, identified through the quadruple helix (based on interest and influence of stakeholders; only highest ranked stakeholders are involved), collaboratively define a problem statement and a vision that outlines solutions to address the identified issues. As a next step, backcasting creates a future narrative that gradually moves backward to identify innovative strategies and actions for the region. This process establishes monitoring needs by considering resilience goals for the future. Living labs facilitate the development of monitoring strategies specific to case study regions. Indicators are developed with the involved stakeholders, ensuring a comprehensive and participatory approach to resilience planning and assessment. Variable case specific technical solutions are applied for data sourcing for monitoring, such as ground sensors, satellite data, drone missions, and citizen science applications, depending on the nature of the monitored variable, the demanded temporal and spatial scales, and technical requirements.

The example of Athens Metropolitan Area case study operates as one of the frontrunners for ARSINOE. The case study focuses on building urban resilience against heatwaves. ARSINOE’s living lab for the Athenian case study has been challenged to elaborate on the Sendai Framework and the risk equation linked to the hazard of heatwaves. Firstly, compound hazards, such as air pollution, biodiversity loss, noise, and violence, are identified through a systemic scanning. Secondly, vulnerable subjects linked to the formed hazard matrix are identified, such as human health—including mental health—well-being, biodiversity, and tourism. Finally, exposure dimensions are attributed to the vulnerability objects. Based on the aforementioned break down analysis of the risk equation a list of indicators is formed including i) hazard indicators (land surface temperature, air temperature, the air quality index), ii) vulnerability indicators (elderly, retired, living in houses built before 1980, living alone, renting, living in houses smaller than 60 sq m, unemployed, immigrants from low and middle income countries—the aforementioned are combined to a Socioeconomic Heat Vulnerability Index), iii) exposure indicators (such as population and population density) and iv) capacity indicators, (trees, green areas, and accessibility to green).

The example of Athens unveils that to some extent, when scoping the problem in a hollistic view, resilience planning and climate resilience are two circles that share a wide overlap, in regards to monitoring and assessing. However they are not the same. Hazard specific indicators for all the parts of the risk equation need to be monitored, while at the same time there are non hazard specific vulnerability and capacity indicators that remain relevant to the specific problem and need to be incuded. Indicative of this differentiation is the monitoring of air temperature, air quality, and the elderly, which are all heatwave specific attributes and linked to the climate resilience monitoring, whereas the unemployed is a non hazard specific attribute, that is linked to resilience planning, yet remains extremely relevant to heatwaves resilience as well.

In regards to the SDGs framework and related indicators, a list of selected SDG indicators is formed of two Tiers, high relevence and moderate relevance. Some of the high relevance SDG indicators are the following: 3.9.1—Mortality rate attributed to household and ambient air pollution, 6.6.1—Change in the extent of water-related ecosystems, 11.5.1—Number of deaths, missing persons and directly affected persons attributed to disasters per 100,000 population, and 15.5.1—Red List Index.

### TransformAr project and its approach to monitoring climate resilience

TransformAr is demonstrating how knowledge and co-innovation processes can drive transformational adaptation towards climate resilience in vulnerable regions and communities across Europe. This involves six demonstrator regions to develop, test, and scale products and services that catalyze significant adaptation efforts. To demonstrate progress towards climate resilience, the steps of the Regional Adaptation Support Tool ( RAST, n.d.) are followed, and thus cover assessing climate risk and vulnerabilities, co-selection of adaptation options and pathways, and evaluating their impact (ex-ante and ex-post), implementation of selected solutions and monitoring, evaluation and learning.

***Monitoring framework***

In TransformAr, the term monitoring is understood broadly and encompasses the following approaches: 1) monitoring is used as the collection of evidences to assess climate risk and vulnerabilities and the impact of adaptation actions (ex-ante and ex-post) and processes, including the characterization of Key Performance Indicators (KPI’s); The collected evidence originates from various sources, including climate risk modelling, expert judgment, and citizen surveys. 2) monitoring is also understood as measuring biophysical parameters in the field. Examples of field monitoring are nutrient and water availability in rural England and urban Finland, coastal and estuarine flow dynamics in Sardinia (Italy) and Galicia (Spain) and urban heat and air quality in peri-urban Egaleo (Greece); 3) As part of a methodological innovation to better grasp and quantify the level of resilience of a system, a resilience index is composed and tested for the aquaculture value chain in Galicia. The resilience index contributes to the typically insufficient understanding of how resilient a system along the whole operational chain is and what can be done to improve it; 4) the achieved outcomes are documented in learning stories. Learning stories are easy-to-read brief documents that digest the findings and thus contribute to the learning component of monitoring. Examples of the application at demonstrators are listed in the paragraph below.

***Monitoring at demo-site level***

For the climate risk and vulnerabilities assessment, CMIP6 climate projections are downscaled and aggregated to NUTS2 level, and consequently translated into sectoral impacts (incl. water, agriculture, fisheries, tourism, urban) using physically based modelling approach. The demonstrator-specific climate risk and vulnerability data have informed the actor-driven selection of adaptation options and pathways; A main challenge was the translation of simulated data to understandable charts tailored to their region. A climate service web platform where the data is available is under development (expected in 2025). The available climate impact data however is often too coarse to assess the ex-ante impact of adaption options and pathways. A hybrid (semi quantitative/qualitative) multi-criteria analysis has thus been used to assess the impact of adaptation options and pathways. The socio-economic aspects of climate adaptation are generated both by macro-economic modelling (e.g. the impact of adaptation on jobs and GDP), but also by bottom-up approach that involves developing indicators at the local level to enhance the granularity of the monitoring process. Additionally, by applying discrete choice experiments and developing a rapid cost-benefit analysis tool, further data is generated to evaluate effectiveness of climate adaptation measures.

Whilst a publication on the resilience index with more detailed information is pending (led by the University of Vigo, Spain), the resilience index is composed by indicators for the following dimensions: governance, research, development and innovation, risk management, collaboration and operational management; The resilience index has been co-created with stakeholders along the Galician aquaculture chain. Delphi panels led to the selection of risk scenarios, indicators and the scoring of indicators.

### IMPETUS project and its approach to monitoring climate resilience

IMPETUS employs Resilience Knowledge Boosters (RKBs) to create scalable and multi-level open knowledge spaces offering opportunities for experts, key communities, and quintuple helix stakeholders to share experiences and knowledge for implementing dynamic pathways and packages for climate adaptation. The IMPETUS RKBs are multidisciplinary communities of actors (the “Human Dimension”) supported by a digital platform (the “Digital Dimension”) designed to enhance regional climate resilience through integrated data, models, and expertise. The RKB platform facilitates stakeholder engagement and co-creation. As a network, it promotes knowledge exchange and enables successful climate adaptation approaches to be shared across communities, ensuring broader and more effective climate resilience strategies.”

***Monitoring framework***

Monitoring is an overarching topic for the IMPETUS project. Within the project, research activities started with a literature review about existing monitoring and indicator frameworks that are relevant for climate change vulnerability and adaptation. The review included the analysis of global frameworks (e.g., the Sendai Framework for Disaster Risk Reduction, the Agenda 2030 and its SDGs, the Lancet Countdown), international frameworks that track adaptation progress at the subnational and city level (C40 ^3^, Covenant of Mayors ^4^, UN New Urban ^5^ Agenda), national frameworks (adaptation plans and strategies) and papers and publications. This work produced a “flexible superset” of indicators (“Metrics for climate change vulnerability, resilience and adaptation”, Koop *et al.*, 2022), that also incorporated the feedback of selected stakeholders. The framework is called *superset*, since it includes a long list of indicators that both cover vulnerability and adaptation issues. It is also referred as *flexible*, since metrics associated to indicators can be tailored to different situations and different data availabilities. Indicators are organized into different categories and subcategories and are searchable by sector and impact. Serving as a structured tool for climate-sensitive decision-making, this framework has acted as an initial indicator repository for several activities of the IMPETUS project. It is expected to be further adapted and revised based on new learnings and context-specificities at local and regional level.

***Monitoring at demo-site level***

Based on the above-mentioned indicator framework, the seven demonstration sites of the IMPETUS project, representing different biogeographical regions, are working to select stakeholder-customizable indicators and metrics that fit their context and eventually add additional site-specific indicators. Selected indicators will feed the IMPETUS Regional Climate Resilience footprint tool, that will allow stakeholders to assess regional resilience and its evolution over time, capturing the effect of adaptation interventions. Within IMPETUS, indicators are seen as tools to assess the current vulnerability state of each demonstration site, predict, verify and continuously reassess the effect of alternative adaptation options and adaptation pathways, supporting flexible and dynamic decision making in the adaptation process. Stakeholders of the seven IMPETUS demonstration sites started to tailor the initially developed indicator framework to their needs, considering a very large variety of climate change risks: flooding, water scarcity, marine storms, fires, biodiversity loss, health diseases, temperature increase, avalanche increase, and extreme storms. Stakeholder engagement is a key action implemented throughout the whole project. It allows to capitalize on local knowledge and thus enable the identification of effective indicators at local scale of the adaptation process.

## Learnings of the projects

In the process of developing their individual monitoring approach, all three projects started off by screening existing and acknowledged global frameworks, such as the Paris Agreement, the Sendai Framework for Disaster Risk Reduction, the Agenda 2030 and its SDGs, the Lancet Countdown, the One Health approach and the Water-Energy-Food Nexus approach.

However, existing global indicator frameworks pose several limitations and challenges that have been widely discussed in several academic and technical papers (e.g. ( Bours *et al.*, 2014; Hammill *et al.*, 2014; Leiter & Olhoff, 2019; Sanchez Martinez *et al.*, 2018; Stadelmann *et al.*, 2015; UNFCCC Adaptation Committee, 2022; Vallejo, 2017). An analysis of these limitations and challenges can be found in the “Metrics for climate change vulnerability, resilience and adaptation” report by IMPETUS ( Koop *et al.*, 2022). The main limitations can be traced back to the following aspects:

- Climate change is global but adaptation takes place on a local level: Indicators and metrics should be site- and context-specific which cannot be reflected in universal frameworks like the aforementioned.
- Interconnectedness of adaptation and vulnerability: Data that is used in global metrics does not reflect local/regional adaptation initiatives and their impacts
- Adaptation monitoring lacks clear targets: Adaptation lacks common measurable targets due to its ongoing and dynamic nature. Unlike mitigation, there is no clear endpoint, making monitoring complex.
- Lack of agreed baseline to assess changes: The absence of a well-defined baseline hinders assessing the impact of interventions, as the overall context is dynamic, requiring more than a simple 'before' and 'after' comparison.
- Complexity in measuring adaptation success: Measuring the success of adaptation is intricate, involving the quantification of "avoided impacts" and dealing with time lags (between intervention and measurable impacts), attribution challenges, and the potential for long-term changes.
- Maladaptation not sufficiently reflected in indicators: Indicators may not adequately signal maladaptation, as they measure adaptation progress but often fail to assess the overall quality, environmental sustainability, and potential negative side-effects.
- Limited explanatory power of indicators: Indicators primarily reflect progress or change but often lack the depth to explain how, why, and what improvements could be made. Understanding the overall adaptation process behind the numerical value expressed by the indicator is crucial.
- Resource- and data-intense nature of monitoring: Monitoring adaptation demands significant resources, including suitable data and technical capacity. Barriers include the lack of long-time series for certain variables, decentralized data, and variations in calculation methods that hinder the uptake of indicators from international frameworks.

There is multiple literature outlining the challenges of monitoring, Within this paper we delve into the early stage and initial challenges and lessons learned derived by the three outlined projects through workshops and interviews during the development of their respective monitoring schemes. These are preliminary learnings as the three projects are ongoing until 2025.

### From global indicator sets to demo-site specific indicator sets and metrics

All three IA projects are following the approach of grounding their monitoring work on existing frameworks and approaches (the ones named above) and tailoring them to demo-site and project specific needs. Climate change affects different biogeographical regions, systems and sectors in diverse ways, whereas the projects are active in various regions and demo-sites. Considering this, it is challenging to provide a comprehensive and exhaustive list of indicators with defined targets that measure resilience. To address this challenge, a number of approaches have proven useful:

- **Create synergies with existing global frameworks**: Existing monitoring systems at the global level (e.g. Sendai Framework ( UNDRR, 2015), SDGs ( United Nations, 2015)), European level (adaptation reporting system for the EU governance of the energy union and climate action ( European Union, 2018) regional, and local level (city networks e.g. C40 cities ( C40 Cities, 2020) provide a robust foundation for planning indicator-based monitoring of climate resilience.
- **Specify indicators:** Using indicators of global frameworks offer comparability across diverse contexts, e.g. SDG indicators, promoting scalability and replicability. However, such a usage of broad, global indicators might fail to account for local/project-specific/regional contexts. Site-specific/biogeographical indicators provide more accurate insights. Projects operating across various contexts and demonstration sites should prioritize a deep understanding of local requirements. This involves engaging experts and stakeholders in an iterative process to select indicators that are relevant and meaningful to each specific location. While customizing monitoring approaches to individual sites or biogeographic regions is advisable, excessive diversity in indicator subsets presents a challenge. This diversity can hinder comparability and impede a comprehensive assessment of project progress and resilience objectives across demo-sites.
- **Prioritize indicators**: Providing a comprehensive and exhaustive list of indicators for monitoring adaptation and vulnerability is extremely challenging due to the different sectors and systems that are affected by climate change in different biogeographical regions. Also, a framework is difficult to apply and adapt when it consists of too many indicators and does not provide guidance on selecting the most relevant ones or on how to tailor them or how to work with data gaps. Thus, it is helpful to start with a smaller number of indicators and build up the set as experience grows.
- **Allocate resources (time and financial)**: Monitoring demands substantial resources within a project, encompassing both time and financial investments. This concerns the phases of the project’s inception and the design of the monitoring approach and ideally extends beyond its implementation. It is crucial to explicitly acknowledge and reflect this reality in project planning document as early on as possible to ensure adequate allocation of resources and realistic expectations regarding monitoring efforts.

### Data for monitoring

As mentioned before, measuring climate resilience can be highly complex. Especially when the aim is to tailor monitoring frameworks to regional and local contexts. It requires substantial amounts of data which covers different temporal and spatial scales to allow long-term monitoring and evaluation:

- **Screen data availability at the start**: Finding easily accessible and suitable datasets for the calculation of certain indicators at regional and local level can be challenging. It is crucial to assess data availability at the regional level early on (ideally right at project start) to avoid developing an indicator framework that might not be put into practice due to data constraints. Specifically, socio-economic data, e.g. data on knowledge and education/financial resources etc. is often not available in a high spatial resolution and is recorded with a lower frequency compared to biophysical attributes (e.g. NUTS3). There is thus a clear need to strengthen data collection endeavors at the beginning of a project. Baseline data plays a critical role in attributing changes to adaptation measures, but its adequacy and accessibility are key.
- **Use qualitative data**: The lack of quantitative (baseline) data is a common challenge, and in such cases, the projects emphasize the use of qualitative data derived from surveys, interviews or via living labs that reflect perceptions of key stakeholders. This can cover social, cultural and political dimensions to climate resilience and increase the visibility of local perspectives. Incorporating narratives alongside quantitative data can provide a more comprehensive understanding of quantitative data, enhance the clarity and context of results, and help to better interpret the outcomes for evaluation, such as ARSINOE, TransformAr and IMPETUS are doing it through their living labs, choice experiments and stakeholder workshops. Additionally, the projects emphasize a stronger inclusion of citizen science data and virtual reality feedback from communities as well as choice experiments.
- **Define a method for harmonizing data**: Approaches and methods for facilitating the harmonization of data are crucial as they enable the translation of diverse data (with e.g. varying formats, differing units, temporal misalignments etc.) into a standardized format. This includes addressing challenges related to units and normalization. Harmonization plays a vital role in aligning indicators and ensuring a shared understanding of data. However, this can pose challenges to projects, especially in trans-disciplinary contexts and should be addressed when gathering different data sources. Moreover, transparency about the methodology employed in the harmonization and aggregation process is crucial. It enables stakeholders to understand how the data was processed, and interpreted, fostering trust and allowing for meaningful analysis and decision-making.
- **The challenge of attribution:** Even if all challenges in data collection have been mastered, more efforts are required to attribute certain changes in indicators to an intervention ( Koop *et al.*, 2022). This is due to the presence of significant time lags between adaptation interventions and measurable impacts, and complex, long-term changes may not be straightforwardly attributed solely to adaptation interventions.

### Interdisciplinarity in climate adaptation

Climate adaptation, by its nature, involves collaboration and expertise from diverse disciplines such as environmental science, social sciences, engineering, economics, and spatial planning. To tackle the intricate challenges of climate change, a comprehensive approach spanning these disciplines is crucial. However, when it comes to monitoring climate adaptation measures, the interdisciplinary nature poses challenges. Effectively integrating diverse perspectives, methodologies, and data sources becomes a hurdle, often resulting in difficulties defining indicators and ensuring comprehensive assessments across multiple disciplines:

- **Define terms clearly**: Whether at the project or demo-site level, clarity in defining key terms like *hazard, exposure, risk, vulnerability, sensitivity, adaptive capacity, and resilience* is paramount both for stakeholders but also for project members. Additionally, it is crucial, right from the project's outset, to ensure a common understanding of terms like *monitoring, evaluation, indicators, metrics, measures, targets, etc.* One authoritative source for definitions is the glossary of the IPCC ( IPCC, 2022). This is an essential starting point for further determining monitoring metrics and indicators from various disciplines. It is thus crucial at the start of a project to determine a common understanding of terms and concepts that are used throughout the project.
- **Foster a shared understanding of different disciplines and sectors**: Achieving a shared understanding across diverse disciplines and sectors, including academia, industry, policy-making, and civil society, presents a challenge in interdisciplinary projects. Members and stakeholders, each rooted in their respective fields, bring varied perceptions and "languages" of monitoring and evaluation, hindering clear communication. Addressing that at project start and finding a mode to integrate diverse expertise, roles, and perspectives is crucial for overcoming these challenges and facilitating effective communication and understanding in the interdisciplinary context. This entails promoting a holistic understanding of the complexity of resilience and adaptation as well as breaking down disciplinary silos. Recognizing that each hazard entails a complex and intricately connected system, the explicit interlinkages are yet to be fully understood and for that, interdisciplinarity is a tremendous advantage.

### Stakeholder engagement

The human dimension of monitoring climate resilience is crucial. Involving stakeholders is essential for selecting the most suitable indicators and identifying existing databases as the specific context and local specificities are best known by stakeholders.

- **Strategically engage stakeholders**: In the projects, different formats of stakeholder engagement were employed, some of which were also used for developing monitoring indicators. A successful approach strategically includes stakeholders identified as high ranking in both influence and interest. The Quadruple and Quintuple Helix approach used in the projects recognize the importance of a broader participation in decision-making processes and innovation activities ( Braun *et al.*, 2021; Carayannis *et al.*, 2012). Not only representatives from government, industry and academia (triple helix, the knowledge of economy) but also the civil society (quadruple helix, the knowledge of society) and the natural environment (quintuple helix, the knowledge of the environment) have been considered in the stakeholder engagement activities of the three projects. The primary objective was to identify positive impacts in terms of adaptation for each demo-site and articulate the essential adaptation pathways and indicators for measuring these impacts. Engagement formats such as workshops, small thematic focus groups addressing technical and policy aspects, and bilateral meetings were used. This flexible and adaptable approach allows for active co-creation with stakeholders, facilitating the identification of tailored indicators aligned with their unique adaptation pathways. Focus group discussions were instrumental in uncovering diverse pathways and narratives. ARSINOE engaged stakeholders through living labs, which supported the monitoring process by contextualizing the monitoring approach to case study regions. IMPETUS engages stakeholders in monitoring resilience and assessing alternative adaptation pathways, through tailoring indicators and metrics to the local level and adding site-specific indicators.
- **Identify blind spots**: Stakeholder engagement proves valuable in discovering previously overlooked aspects of climate resilience. Subsystems that were not initially recognized or considered important are revealed through stakeholders’ hints, especially through identifying interlinkages of vulnerabilities, compound hazards and cascading risks. Incorporating stakeholder input adds nuance and depth to the identification of monitoring priorities, contributing to the development of a comprehensive list of indicators. Within the projects, different engagement processes were utilized. ARSINOE opted for living labs, where stakeholders collaborated to shape a shared vision for a climate-adapted future. In these settings, stakeholders pinpointed critical subsystems crucial for building resilience in e.g. a demo-site regarding a specific hazard – thereby helping to determine the most relevant indicators. This approach ensures that indicators align with the practical needs and priorities of those directly affected. IMPETUS used participatory methods for exploiting the knowledge of different disciplines and backgrounds of stakeholders to develop site-specific indicators for monitoring. The result is an enhancement in the robustness and relevance of the entire monitoring and adaptation process.
- **Generate qualitative data**: Moreover, stakeholders play a key role in providing qualitative data and narratives, contributing to a holistic understanding of adaptation efforts that goes beyond quantitative data, e.g. grounded in a theory of change. Transformar for example employed choice experiments and a rapid cost-benefit-analysis tool that provides qualitative data which can be used for monitoring. IMPETUS encourages the use of qualitative information to shape adaptation pathways in demonstration sites, whenever numerical data or modeling capacity are lacking.

## Conclusion and outlook

The approaches of the three projects on monitoring climate resilience have unveiled challenges and insights that other projects can learn from. Monitoring climate adaptation measures poses a unique challenge due to the interdisciplinary nature of the field, requiring effective integration of diverse perspectives, methodologies, and data sources, with key considerations including the clear definition of terms, the integration of different disciplines for shared understanding among all project members and stakeholders. Basing the development of indicators on existing global frameworks is advised, together with the contextualization of indicators to local needs. Accessible and available (baseline) data forms a fundamental element of this preparation. Equally critical is the implementation of a well-structured stakeholder engagement process from the project's inception, ensuring diverse perspectives and insights are considered. Additionally, proactive measures should be in place to guarantee the continuity of monitoring (and evaluation) efforts even after the project concludes.

It is recommended that the insights gained here serve as a catalyst for a wider dialogue within the Mission Implementation Forum, fostering a community of practice and extending beyond individual projects to engage the broader regional community in establishing effective monitoring and evaluation practices.

## Data Availability

No data are associated with this article.

## Footnotes

^1^ SustainGraph is a knowledge graph toolwhich acts as ARSINOE’s unified knowledge source, leveraging graph databases and machine learning techniques for data population, knowledge production, and analysis. It maximizes the use of available data, ensuring openness and interoperability with existing databases and Application Programming Interfaces. It implements the channeling of such data into models and unveils hidden relationships and/or patterns. ( Fotopoulou *et al*., 2022)

^2^ Systems Innovation Approach is a holistic method, adapted by ARSINOE, for addressing the complex and adaptive problem of increasing resilience against climate change and compound challenges, by understanding the interconnectedness of the elements within the involved systems. Unlike traditional approaches that might focus on individual components, in an analytical manner, systems innovation looks at the system as a whole, acknowledging that changes in one of the components might affect the entire system.

^3^ https://resourcecentre.c40.org/resources/monitoring-evaluating-and-reporting

^4^ https://www.eumayors.eu/support/adaptation-resources.html

^5^ https://www.urbanagendaplatform.org/data_analytics
