# Supplementary material for: Deriving lessons learned from monitoring adaptation activities in projects under the EU mission on adaptation
Source: Open Res Eur. 2025 Nov 12;4:81. Originally published 2024 Apr 24. [Version 2] doi: 10.12688/openreseurope.17372.2 (PMC11185904; doi:10.12688/openreseurope.17372.2)
Supplement: Peer review history

# Reviewer report: Reviewer response for version 1

**DOI:** 10.21956/openreseurope.18775.r41279

**Published:** 2024-07-05

**Authors:** Tom Mitchell, Sanchita Bakshi

**Affiliations:** 1 International Institute for Environment and Development, London, England, UK; 2 IIED Europe, Amsterdam, Netherlands, The Netherlands

Dear authors, many thanks for giving us an opportunity to review this important paper. It is an insightful work that draws on lessons learned from three EU Horizon 2020 projects under the EU Mission on Climate Adaptation to Climate Change. We propose the following few items for your consideration to improve the overall quality of the submission.

 1. The authors correctly highlight that monitoring and evaluation (M&E) serve multiple purposes in adaptation projects. In a cyclical policy and agenda-setting process, insights from M&E provide crucial knowledge for shaping and refining adaptation policies and measures. Understanding what works, under what conditions, and why, empowers regions to enhance their adaptation policies. M & E also builds accountability, especially towards the most vulnerable. It ensures that actions are equitable and support vulnerable communities without unintentionally exacerbating inequalities (Lager et al. 2023). Additionally, effective M&E can enhance our understanding of evolving climate risks. By bridging knowledge gaps and illuminating critical challenges and opportunities, M&E lays a robust groundwork for effective adaptation planning (Leitner et al. 2020). The authors acknowledge many of these versatile purposes of M&E for effective adaptation. However, the discussion misses some aspects of the just nature of adaptation interventions, a minor omission that can be easily addressed.

 2.The authors also recognize the challenges of monitoring adaptation, such as the lack of a standardised framework and the involvement of multiple sectors. However, the discussion lacks consideration of the different timeframes, often long-term nature of adaptation projects, as also the attribution challenges inherent to these projects. Incorporating these elements will provide a comprehensive and complete overview of the discussion on monitoring and evaluation in adaptation.

 3.The methodology and the thematic analysis in the introduction are somewhat indistinct. While these elements are not always easy to separate, a bit more attempt to distinguish between the manner in which the 4 analytical themes were identified and what the 4 themes claim, would provide more bite and clarity to the current paper. Additionally, the introduction could be structured slightly differently to highlight what makes the 3 projects particularly interesting to study MEL for adaptation.

 4.Currently, the paper focuses heavily on methodology, with less attention given to identifying an interesting puzzle/ research question from the three projects. The authors may like to frame the four thematic explorations from the expert group workshop as specific crosscutting issues within monitoring adaptation that genuinely demand explanation, rather than presenting them as general phenomena requiring sophisticated description.

 Reframing the introduction in this way will engage the reader's curiosity by presenting issues that need to be explained rather than merely described.

 5.It is difficult to comment on the 3 projects as these are large, complex, involve creative collaborations, and are currently ongoing. However, more empirical detail about the monitoring tools, techniques, and frameworks used in the projects is needed and would add the necessary specificity to the analysis. For instance, in discussing the ARSINOE project, the reviewer is interested in the indicators used for resilience planning and assessment. Additionally, it is unclear whether the authors suggest that monitoring and assessing resilience planning is the same as monitoring and assessing climate resilience. If not, the exact focus of the monitoring remains unclear from the text. Similarly, in the description of the monitoring approach in TransformAr project, the nature of the new data generated is not clearly explained. The discussion on the IMPETUS project is interesting but does not tell us what is novel about the approach adopted. More detailed information would help clarify these points.

 6.A brief explanation, perhaps in the footnotes or endnotes, of terms such as Systems Innovation Approach, and Resilience Knowledge Boosters (RKB) would be helpful.

 7.The section on global frameworks is slightly misleading because it includes challenges of monitoring adaptation that extend beyond global frameworks. A subheading or a separate heading for better sign-posting would improve clarity.

 8.It would be helpful to know whether and to what extent the monitoring approaches of the 3 EU projects have addressed some of the challenges listed by the authors. Currently, the discussion on the monitoring approaches adopted by the three EU projects is separate from the overall context of the constraints in monitoring adaptation. Adding a paragraph linking the two would be beneficial. Alternatively, a simple table format could be used to show whether and to what extent the limitations listed by the authors have been addressed in the 3 EU projects. This will also nicely connect to the final section on learnings, where proposed solutions and policy advice emerging from the 3 EU projects are presented.

Where applicable, are recommendations and next steps explained clearly for others to follow? (Please consider whether others in the research community would be able to implement guidelines or recommendations and/or constructively engage in the debate)

Yes

Does the article adequately reference differing views and opinions?

Yes

Are all factual statements correct, and are statements and arguments made adequately supported by citations?

Yes

Is the rationale for the Open Letter provided in sufficient detail? (Please consider whether existing challenges in the field are outlined clearly and whether the purpose of the letter is explained)

Yes

Is the Open Letter written in accessible language? (Please consider whether all subject-specific terms, concepts and abbreviations are explained)

Yes

Reviewer Expertise:

Climate change adaptation monitoring and evaluation approaches.

We confirm that we have read this submission and believe that we have an appropriate level of expertise to confirm that it is of an acceptable scientific standard, however we have significant reservations, as outlined above.

# Reviewer report: Reviewer response for version 1

**DOI:** 10.21956/openreseurope.18775.r41284

**Published:** 2024-06-18

**Authors:** Emma Tompkins

**Affiliations:** 1 Southampton University, Southampton, UK

The paper reviews the lessons learned by 3 EU Horizon 2020 projects in trying to monitor adaptation.

 There is a little internal inconsistency of aim which needs some small tweaks to resolve. Is this paper about (i) monitoring the process and practice of adaptation, i.e. seeing what adaptation looks like in practice as it unfolds as the title infers, and as is stated in the introduction (“ *monitor and evaluate adaptation actions taken*”).  Other examples of researchers doing this are:[3]. Science and policy lessons learned from a decade of adaptation to the emergent risk of sargassum proliferation across the tropical Atlantic. [3] – shows the process of adaptation over a 10 year period), Or, is this paper about (ii) monitoring the “ *development towards climate resilience*” i.e. how societies can be better prepared to live with climate change. Or, is it about (iii) identifying the challenges in monitoring adaptation – which is the focus of most of the rest of the paper. These are all worthy aims but they are different aims. Some clarification of text in the introduction (and title) is needed to focus on just one of these aims, I assume it will be in the challenges of monitoring. Reflection on the others could be made in the conclusions section perhaps?

 The abstract is very brief and does not really communicate the aim or content of the paper. I would use a more formal type of abstract, i.e. content should include: what is the topic of interest, what is the research gap, what is the aim of the paper, what are the methods and materials, and what are the key findings. At present the abstract suggests that the methods involved workshops with experts – after reading the paper this does not appear to be the case, instead, the paper appears to be a review of 3 projects. If workshops were used to gather data from those running the projects this needs to be explained somewhere. If this is a literature review of the outputs from the projects – that should be explained. Greater clarity in the abstract would help.

 The paper is broadly drawing on lessons from projects which are in progress. I understand the desire to produce fast-track outputs from research, but I would ask is it too early to be evaluating projects that have not yet finished? The four-year long projects started October 2021-March 2022. At this point (June 2024) they will be about 2.5 years into their research which usually would not enable much detailed analysis of progress or findings. Given the typical timing of most large consortium research projects (i.e. y1 getting started/gathering data, y2, more data collection and preliminary analysis, y3, detailed analysis and preliminary findings published; y4+, analysis written up and published), I am unclear how key findings on monitoring progress on adaptation, or progress towards developing climate resilience can be gleaned from these projects at this midway stage.

 In terms of readability of the paper it would have been good to add in some footnotes or endnotes showing the timing of the three projects i.e. 4 years starting in xxx date. Difficult jargon could be better explained at first use e.g. quintuple helix stakeholders.

 It is difficult to critique the sections on the 3 EU projects, as these are vast, early stage, large consortium projects. There is some massaging of evidence to fit to the desired focus of this paper. For example, the ARSINOE project is measuring progress towards the SDG progress, there is no evidence that progress towards the SDGs equates to progress towards climate resilience (“The SustainGraph is a knowledge graph that monitors resilience on a broader level – to the extent where resilience relates to sustainable development – and monitors progress towards achieving SDG-targets”). I am not sure if ARSINOE has or will undertake work to assess whether SDGs are accurate indicators of climate resilience? If it has, this text needs to be added. If not, the text aligning progress on SDGs with progress on climate resilience needs to be changed.  

 The IMPETUS project looks like it will develop interesting information on adaptation. It proposes to develop metrics of climate vulnerability and adaptation. The vulnerability metrics look very similar to the work of Brooks et al, 2005 (“The determinants of vulnerability and adaptive capacity at the national level and the implications for adaptation”). The adaptation metrics follow the commonly used approach of monitoring government delivery of institutional adaptation initiatives.  I am unclear how these are taking forward our understanding of monitoring adaptation (beyond[1],[2] ). Documenting the state of adaptation for the global stocktake of the Paris Agreement. Wiley Interdisciplinary Reviews: Climate Change, 9,5, e545.). Some additional text explaining clearly to reader the additional contribution made by this work would be useful.

 The section on global frameworks offers some reflection on the limitations of monitoring adaptation, however most of the limitations listed could be gleaned from extant academic sources on the challenges of monitoring adaptation (see[1]etc.. above).

Where applicable, are recommendations and next steps explained clearly for others to follow? (Please consider whether others in the research community would be able to implement guidelines or recommendations and/or constructively engage in the debate)

Partly

Does the article adequately reference differing views and opinions?

Partly

Are all factual statements correct, and are statements and arguments made adequately supported by citations?

Partly

Is the rationale for the Open Letter provided in sufficient detail? (Please consider whether existing challenges in the field are outlined clearly and whether the purpose of the letter is explained)

Partly

Is the Open Letter written in accessible language? (Please consider whether all subject-specific terms, concepts and abbreviations are explained)

Partly

Reviewer Expertise:

climate change adaptation

I confirm that I have read this submission and believe that I have an appropriate level of expertise to confirm that it is of an acceptable scientific standard, however I have significant reservations, as outlined above.

## References

[1] : Tracking global climate change adaptation among governments. *Nature Climate Change* .2019;9(6) : 10.1038/s41558-019-0490-0 440-449 10.1038/s41558-019-0490-0

[2] : Documenting the state of adaptation for the global stocktake of the Paris Agreement. *WIREs Climate Change* .2018;9(5) : 10.1002/wcc.545 10.1002/wcc.545

[3] : Science and policy lessons learned from a decade of adaptation to the emergent risk of sargassum proliferation across the tropical Atlantic. *Environmental Research Communications* .2023;5(6) : 10.1088/2515-7620/acd493 10.1088/2515-7620/acd493

[4] : The determinants of vulnerability and adaptive capacity at the national level and the implications for adaptation. *Global Environmental Change* .2005;15(2) : 10.1016/j.gloenvcha.2004.12.006 151-163 10.1016/j.gloenvcha.2004.12.006

# Reviewer report: Reviewer response for version 1

**DOI:** 10.21956/openreseurope.18775.r40827

**Published:** 2024-06-17

**Authors:** Diana Reckien

**Affiliations:** 1 University of Twente, Enschede, The Netherlands

Dear Authors,  thank you very much for this insightful paper. This is a timely topic and studies on the lessons learned after implementing climate responses are crucial.     

 I have a few suggestions how to improve the paper:

 - in the abstract, you mention that the workshop yielded insights into "four themes." This is raising a question mark. Could you please be more specific: do these four themes relate to challenges or "insights" in a positive way? 

 - in the third paragraph of the article, you refer to the RAST system and the difficulties of applying a generic framework to different regional contexts. The reference that you cite, "Prinlge, 2011," is rather old. In that regard, please include more recent references, e.g. look at the latest IPCC Report AR6 WGII Chapter 17, there are particular sections on monitoring and evaluation as well as a Box that  discusses the difficulties of tracking progress. In these parts of the IPCC AR6 WGII you find a lot of more recent references, including the ones by Leiter et al.

 - Before you can move to the fourth paragraph in the introduction, you could provide more insights into how you arrived at these four topics. It sounds like these were coming out of your workshops; hence, I would report those as results. But in this case, the wording here in the introduction needs to be changed. In the introduction, it sounds like you derived these four themes from a literature review. For these four themes to work as a synthesis of the literature, you need to provide more background/ literature review. This is currently missing. So, either add that or only report these as an outcome of the workshops in the results'section.

 - After the introduction, you go into presenting the projects and then into what I would call "results," i.e., by comparing the projects and their approaches, frameworks, etc., of monitoring. In the abstract, however, you mention that workshops were held. I suggest adding a small section on "Methodology" to get a better overview of how you assessed these projects, why you selected these three, what kind of indicators you choose to compare these projects and why, who joined these workshops, how many people were there, what was discussed at these workshops, etc.

 - I regard the section on "Global Frameworks" as a bit superficial, i.e., it does not go deep enough to explain why these frameworks were chosen and which indicators were useful or not useful. On the other hand, this section is not needed for the article topic. All the information provided in this section can be looked up elsewhere. I would shorten this section substantially, i.e. it is enough just to state the projects used these different frameworks--the current additional text is not needed or would need to be more detailed to be helpful.

 - The issue "Interconnectedness of adaptation and vulnerability: Data that are used in global metrics does not reflect local/regional adaptation initiatives and their impacts" has issues: the information before the ":" and after it do not correspond to each other. Please provide an alternative description after the ":"

 - in the section "Global Frameworks", para #3, sentence #2 there is a verb missing. Rephrase. 

 - in the section "Learnings of the project" you repeat a lot of the information that is given in earlier sections, e.g. in the global frameworks section. I would shorten this section to avoid redundancies. E.g., the bolded keywords in the beginning of each item in the list (e.g. "Specify indicators"; "prioritize indicators") is a good take-away/ a learning from the project, but what you provide as description and explanation after that (not bold) is in parts a repetition with earlier text. 

 - the text after "evaluation of data" is not related to learning. It describes the challenges but not the solution. In relation to my previous comment, it would be helpful if you keep the focus on the learning and not on the problem. E.g. it would be helpful to know whether you derived those "learnings" from reviewing data/ workshop notes or whether these were mentioned by the workshop participants themselves. How did you get to these learnings is a general question that I have. This question will need to be answered to assess the validity of your findings. 

 - being a bit more critical and detailed to the learnings as presented in the section "stakeholder engagement" is particularly needed, as this is usual a critical point. I.e., how do you strategically engage stakeholders? Was that done successfully in any of these projects?  Who said that? 

 - general, I am asking myself whether all these learning come out of all three projects assessed or only two of them? How do you derive your findings/ results?

 - in the section on "identifying blind spots" you mention "some" which raises the question who these 'some' are? Please can you be more specific. 

  - in general, in this section on stakeholders, please bring examples, i.e. who exactly said what? E.g. "different engagement processes were utilized". this is a very generic sentence and needs further specification to be useful as a result/ learning. "some" and "others" sounds like you had a lot of feedback. But you are only reporting from 3 projects. So, who are "some" and "others"?

 -  The section on "generate qualitative data" is insufficiently detailed. For example, what kind of qualitative data can be used? The description provided re "engagement formats" does not prove why qualitative data is important; these formats could also be used to collect quantitative data. When saying, "focus groups were instrumental ..." the readers asked him/herself why. Please provide your reasoning, e.g. by providing examples. How did you get to this conclusion?

Where applicable, are recommendations and next steps explained clearly for others to follow? (Please consider whether others in the research community would be able to implement guidelines or recommendations and/or constructively engage in the debate)

Partly

Does the article adequately reference differing views and opinions?

Partly

Are all factual statements correct, and are statements and arguments made adequately supported by citations?

Yes

Is the rationale for the Open Letter provided in sufficient detail? (Please consider whether existing challenges in the field are outlined clearly and whether the purpose of the letter is explained)

Partly

Is the Open Letter written in accessible language? (Please consider whether all subject-specific terms, concepts and abbreviations are explained)

Yes

Reviewer Expertise:

Climate change impacts; climate change adaptation; adaptation planning, success; adaptation monitoring & evaluation.

I confirm that I have read this submission and believe that I have an appropriate level of expertise to confirm that it is of an acceptable scientific standard, however I have significant reservations, as outlined above.

# Reviewer report: Reviewer response for version 1

**DOI:** 10.21956/openreseurope.18775.r41280

**Published:** 2024-06-14

**Authors:** Joshua Garland

**Affiliations:** 1 LUCSUS (Lund University Centre for Sustainability Studies), Lund University, Lund, Sweden

This Open Letter, entitled ‘Deriving lessons learned from monitoring adaptation activities in projects under the EU mission on adaptation’, focuses on climate adaptation monitoring as an important challenge in evaluating the outcomes of adaptation efforts. It aims to inform approaches to this based on learning from three ongoing Horizon 2020-funded projects: ARSINOE; TransformAr; IMPETUS. The insights provided emerged through expert workshops concerning monitoring experiences and centre on four central areas: local relevancy; data; interdisciplinarity; stakeholder engagement.

 The Letter’s intention to inform through an account of challenges encountered, and to support learning that benefits monitoring practices, appears to be well-fulfilled. Notable within this is the centrality given to multi-stakeholder engagement, both top-down and bottom-up, to develop location-specific indicators through which to monitor adaptation projects. This is true of each project presented.

 A key argument conveyed within the Letter is the importance of qualitative data. This, it is suggested, can be particularly useful when quantitative (baseline) data are lacking through providing insights into the local or regional socio-cultural and political contexts around adaptation projects (‘Data for monitoring’ section). This also seems appropriate and useful.

 There remain a few additional observations, and some minor suggestions, to make.

 Firstly, some of the terminology could be clarified for greater accessibility for readers who may be uncertain of what certain phrases mean. This is perhaps the case with mention of the quadruple and quintuple helix when introducing the ARSINOE project. These terms are clarified within the penultimate ‘Stakeholder engagement’ section, but could be helpfully defined earlier in relation to ARSINOE. Similar stands for the Systems Innovation Approach principles that could be briefly outlined for added clarity (also in the ARSINOE section). The abbreviation ‘IA’ could be clarified on first usage, too (Global frameworks section). Such definitional work has been well-captured within other parts of the Letter, including around IMPETUS’ ‘Resilience Knowledge Boosters’ and throughout the Global frameworks section.

 The latter presents a very clear and succinct account of international agreements and frameworks, such as the Paris Agreement, while being up-to-date through reference to COP28 outcomes relevant to adaptation. Could there be more to say here about the EU Strategy on Adaptation to Climate Change? Currently the Letter uses this to underline disaster risk and adaptation linkages, but perhaps consideration could be extended by noting some of its core points within the wider EU Green Deal context. These include, for instance, the significance of monitoring and existing data availability limitations that speak well to the Letter’s reflections.

 The Strategy also discussed improving monitoring through developing a ‘harmonised framework of standards and indicators’ (EC (2021) Strategy on Adaptation to Climate Change, section 8) – this is something the Letter and the three projects can directly engage with in a meaningful, practical way through their learning points and suggestions. This is perhaps most clear in the Letter’s treatment of global indicators (Learnings of the project section) in which they are complemented for their comparability while stressing a need for clear harmonisation methodologies and local-specific indicators that could be more insightful; key and useful suggestions.

 Overall, the account of these frameworks’ limitations is also a positive and this Letter does well to summarise some key adaptation challenges in an accessible way, inclusive of maladaptation that could be important for future learning and monitoring activities. By extension, this discussion helps to underline the possible impact of the projects and related reflections.

 It was interesting to read about the living labs and their use would seem appropriate where the purpose is to develop locally-relevant and/or shared monitoring indicators with a range of stakeholders. This is a collaborative approach that could certainly be of use for future projects and initiatives, including those aiming to achieve a bottom-up component. As a result, it would perhaps be beneficial for additional information to be provided, particularly in terms of the practical experiences of the labs gained through the projects. For instance, how many stakeholders were represented, how many labs were held, for how long did they last and were there any challenges encountered through these in terms of participation or similar? Reflections along these lines – even if only brief at this time – may further help others in thinking about, designing and perhaps implementing such an approach that holds important potential to support adaptation monitoring plans.

 Regarding the ARSINOE living labs, it is mentioned that stakeholders were identified through the quadruple helix ‘based on [their] interest and influence’ (first paragraph, Demo-site level monitoring section). It notes also that only the ‘highest ranked’ stakeholders were included. Maybe these could be elaborated upon through adding detail about what criteria was used to define a stakeholder as high-ranked, what qualifies as a relevant interest and how influence was understood and observed in practice. Living labs are nonetheless well-discussed as an approach later in the Letter, in the Stakeholder engagement section.

 It may be useful to know more about the project contexts regarding what kinds of physical environments (natural and/or built) are being focused on, including the type of adaptation projects that are to be monitored. For instance, is the focus on coastal adaptation to erosion and/or flood risk in Europe, or a different adaptation challenge elsewhere? Whether the projects relate to hard and/or soft adaptation options and similar details may help enhance understandings of the contexts from which the Letter’s important insights are drawn.

 In the ‘Data for monitoring’ section it is noted how time lag can pose a challenge to adaptation outcome evaluation, but is there anything from the projects that could point towards possibly fruitful ways of addressing this? Maybe approaches complementary to (quasi-)experimental methods could be of value here? This would be interesting to hear slightly more about as part of the reflections and lesson-learning offered, including in relation to qualitative data.

 Another keen insight concerns the importance of a clear use of terminology and shared understandings of key concepts in multi-stakeholder and multi-disciplinary settings. This is correctly underlined since the vocabulary can be used to emphasise different values and factors, reflecting also prior experiences and knowledge. Such differences may come, for instance, in understanding vulnerability in terms of economic loss or less tangible place attachments, or differences between qualitative and quantitative approaches to measurement and evaluation. Achieving a clear, collective set of definitions is therefore among the key suggestions presented by the Letter.

 Indeed, the stated intention of this Letter is to present learning points from across the three projects and this appears to be well-done. The discussion, including the limitations and suggestions covered, seems both relevant and important to monitoring and impact evaluation questions in the climate adaptation arena. This Letter therefore represents a clear and meaningful contribution that can begin to help guide how future adaptation projects think about monitoring, especially regarding multi-stakeholder and qualitative-quantitative approaches to data and indicator development that are more site-specific and, perhaps, useful in practice. It will certainly be interesting to read more about the outcomes of the three projects as they continue to near completion.

Where applicable, are recommendations and next steps explained clearly for others to follow? (Please consider whether others in the research community would be able to implement guidelines or recommendations and/or constructively engage in the debate)

Yes

Does the article adequately reference differing views and opinions?

Yes

Are all factual statements correct, and are statements and arguments made adequately supported by citations?

Yes

Is the rationale for the Open Letter provided in sufficient detail? (Please consider whether existing challenges in the field are outlined clearly and whether the purpose of the letter is explained)

Yes

Is the Open Letter written in accessible language? (Please consider whether all subject-specific terms, concepts and abbreviations are explained)

Partly

Reviewer Expertise:

Environmental and Climate Governance, including Adaptation; Civil Society; Social Science Methods

I confirm that I have read this submission and believe that I have an appropriate level of expertise to confirm that it is of an acceptable scientific standard.
